# Spatial fishing restrictions benefit demersal stocks in the northeastern Mediterranean Sea

**DOI:** 10.1038/s41598-018-24468-y

**Published:** 2018-04-13

**Authors:** Donna Dimarchopoulou, Aikaterini Dogrammatzi, Paraskevi K. Karachle, Athanassios C. Tsikliras

**Affiliations:** 1 Laboratory of Ichthyology, Department of Zoology, School of Biology, Aristotle University of Thessaloniki, 54124 Thessaloniki, Greece; 2 Institute of Marine Biological Resources and Inland Waters, Hellenic Centre for Marine Research, 16604 Attica, Greece

## Abstract

The multi-level benefits that marine organisms gain when protected from fishing are well acknowledged. Here, we investigated the effects of a 40-year trawling ban on the status of targeted and non-targeted marine species within a major fishing ground in the northeastern Mediterranean Sea (Thermaikos Gulf, Aegean Sea). Biomass and somatic length of fish and invertebrates (six commercial and three non-commercial demersal species) were measured in three areas of varying fishing pressure, depending on the temporal and spatial operational regimes of fishing vessels. The positive effects of fishing restrictions on the studied demersal stocks were clearly revealed, as the commercial fish species exhibited higher biomass in the intermediate and low pressure areas, as well as increasing maximum and mean total length (and other length indicators) with decreasing fishing effort. The mean total length of non-commercial species generally did not differ among areas, except for species caught and discarded at high rates. The present study shows that fishing does alter the population structure and biomass of commercial demersal species, and that fishing restrictions greatly contribute to improving the status of demersal populations within the restricted areas by providing a refuge for large individuals and their important contribution to the gene pool.

## Introduction

Ever since the pioneering work of Fulton^1^ on the effects of trawling on the biomass of marine populations by comparing the catches between areas open and closed to commercial fishing, the issue of the impact of fishing on marine populations^2^ and the beneficial role of fishing restrictions on commercial fishes and invertebrates has remained an integral part of fisheries science^3^. Although the effectiveness of protective restrictions for fisheries management purposes has been questioned^4^, numerous studies conducted in areas of varying protection regimes clearly demonstrate the positive effects of fishing restrictions on preserving fish abundance and biomass, as well as length structure in the Mediterranean Sea^5,6^ and other areas of the world^7,8^.

Fisheries Restricted Areas (FRAs) are geographically distinct areas where fishing is regulated through temporary or permanent closures and bans of certain fishing gears, such as bottom trawls, purse-seines and other gears^9^. FRAs can contribute to biodiversity conservation as complementary structures to conventional Marine Protected Areas (MPAs), even though their primary target might not be conservational *per se* (i.e. conserving ecosystems as a whole, protecting species from extinction, maintaining their habitats as natural and undisturbed as possible, and preserving population structures as well as genetic diversity) but rather focus on maintaining or improving the status of particular stocks and enhancing the respective fisheries^10^. The permanent closure of marine areas to fishing, especially in the coastal zone where spawning and nursery habitats of many species are located^11^, is a practice widely applied for the protection and recovery of fish stocks^12^ and has significant positive effects on spawning stock biomass and recruitment^13^. Besides ecosystem and habitat protection^14^, any spatial fishing restrictions that protect part of the commercial fish and invertebrate populations have been proven to be beneficial because they may lead to biomass and reproductive potential increases inside the protected area, but may also profit adjacent areas through the emigration of juveniles and adults^15^. Apart from the potential effect on target species, fishing refuges can also have a positive impact on community ecology through the protection of species diversity, habitat structure and community stability^16^.

The Mediterranean Sea living resources have been exploited since ancient times^17^. The long-lasting intense fishing pressure applied on the Mediterranean fish and invertebrate stocks has led to declining population biomasses^18^, which is reflected in catches that are also declining for the majority of stocks^19^. The vast majority of Mediterranean fisheries are not managed by total allowable catches and quotas as generally practiced in northern Europe, but by input controls only, i.e. fishing effort management through controls of vessel number, size and allowed fishing time, as well as temporal, seasonal or permanent area closures^20^.

**Figure 1:**
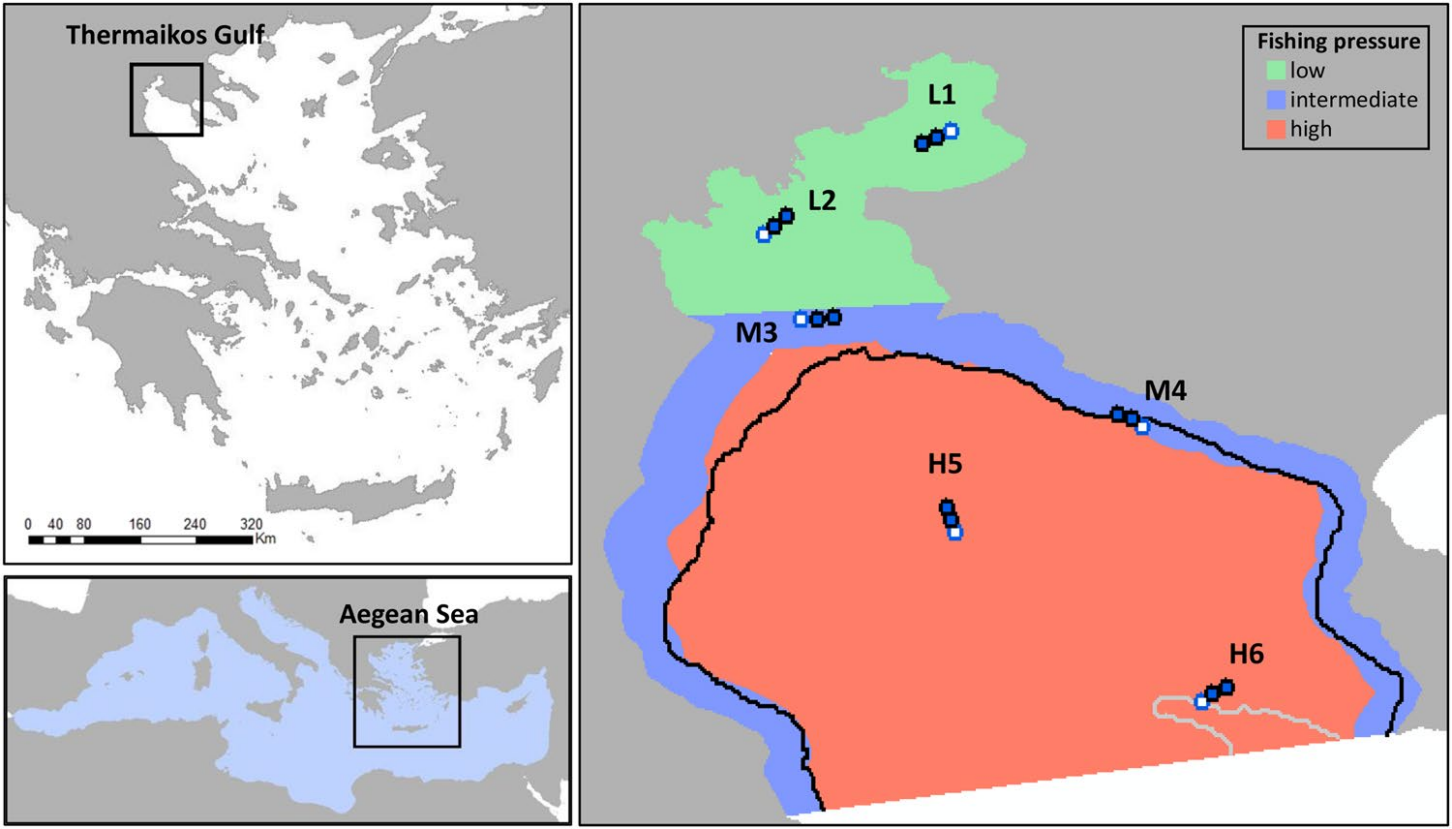
Map of the study area (Thermaikos Gulf; NW Aegean Sea; NE Mediterranean Sea) showing the sampling stations (Hauls 1–6; white circle indicates the starting point of the haul) and the fisheries restricted areas (green: low fishing pressure; blue: intermediate fishing pressure; red: high fishing pressure). Τhe 50 and 100 m depth contours are shown in black and grey, respectively. The maps were generated using ArcGIS 10.4.1.5686 (www.esri.com).

The number of studies on the benefits of MPAs and FRAs on the living resources is rather limited in the eastern sub-region compared to the western and central Mediterranean areas^21,22^, as is the case with most biological characteristics of fishes that are also less studied in the eastern Mediterranean^23^. Therefore, the general objective of this study was to test the hypothesis that fishing restrictions maintain the size structure and protect the biomass of demersal marine populations in Thermaikos Gulf (northeastern Mediterranean Sea, Fig. 1). In order to assess the effectiveness of fishing restrictions in Thermaikos Gulf, we sampled three areas of varying restriction regimes that result in varying fishing pressures, and compared the biomass, length structure and length at maturity of commercially important demersal fish and invertebrate species using various length-based indicators, among these areas. Length is considered as a robust proxy for evaluating the status of a fish population because intensive exploitation selectively removes the older and larger individuals^24^ and reduces the probability of juvenile fish surviving to larger sizes^25^. To corroborate that the observed patterns are attributed to fishing pressure and the imposed fishing restrictions rather than site-specific impacts or random effects, we also included non-commercial demersal fishes in our analyses.

## Results

### Size structure

**Figure 2:**
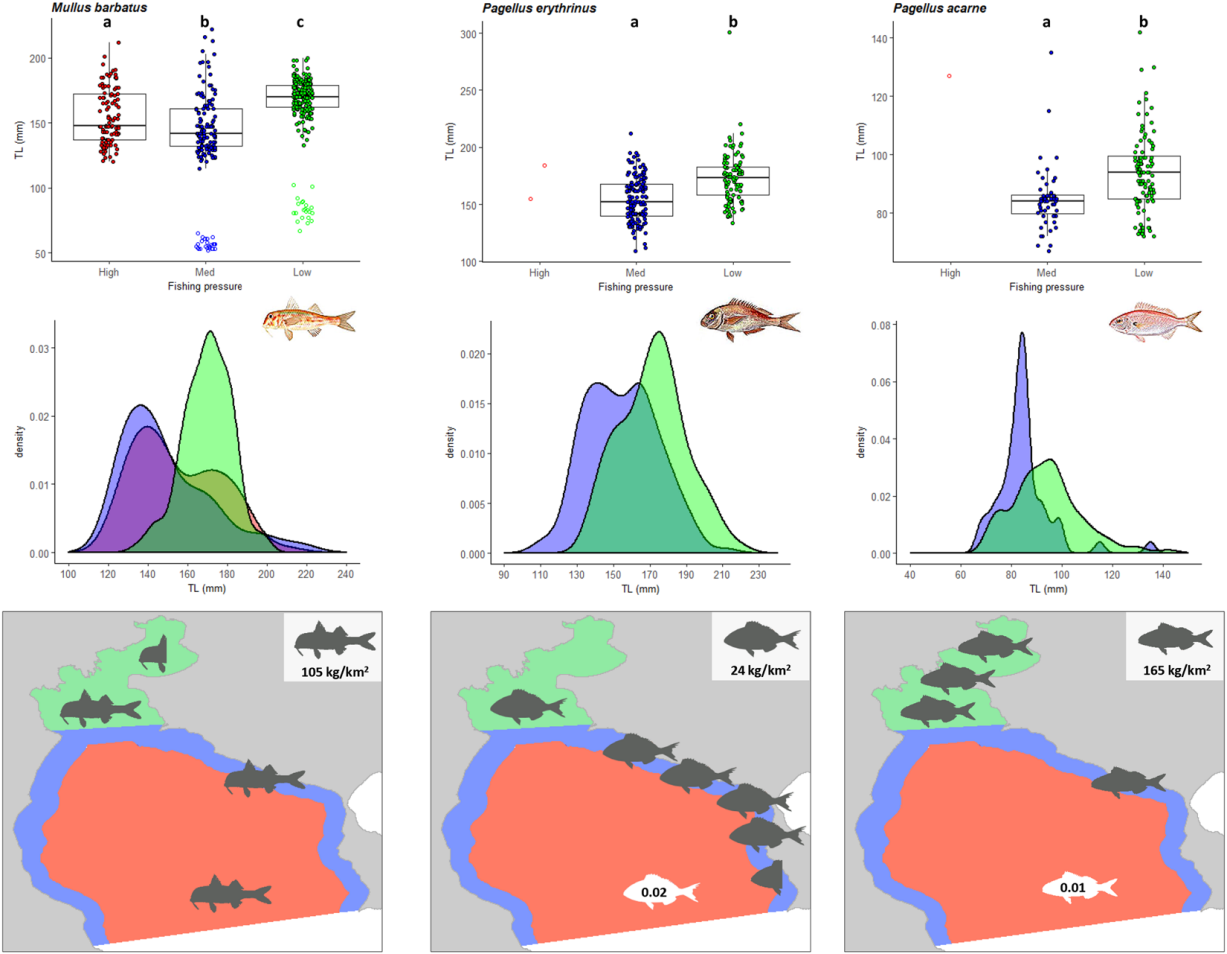
Box plots (total length, mm), density plots of length distribution, and CPUE (kg/km^2^) distribution maps of commercially important demersal fishes (red mullet *Mullus barbatus*, common pandora *Pagellus erythrinus*, axillary seabream *Pagellus acarne*). The maps were generated using ArcGIS 10.4.1.5686 (www.esri.com). Fish drawings are by Louizos Verdaris.

**Figure 3:**
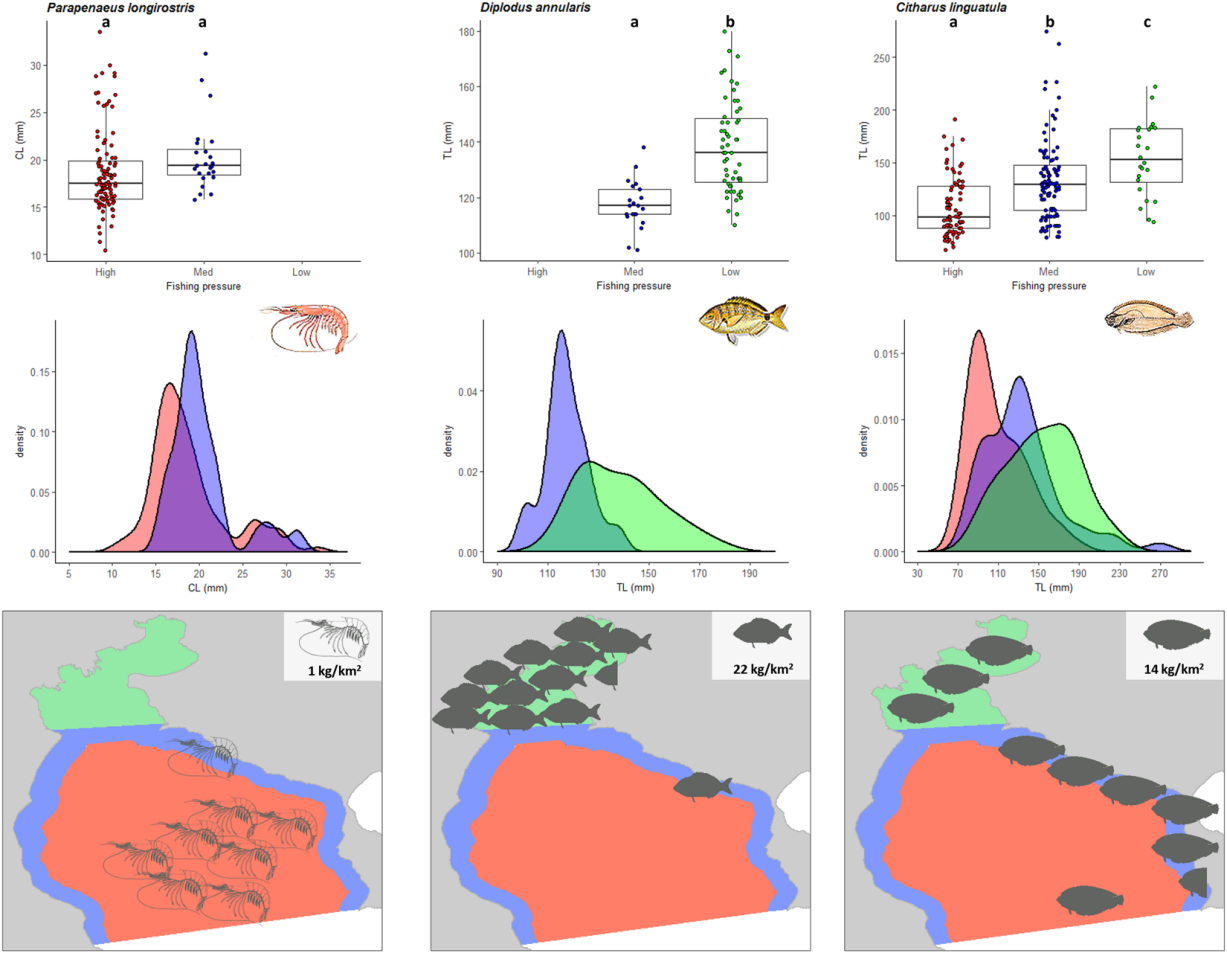
Box plots (total length, mm for fishes; carapace length, mm for invertebrates), density plots of length distribution, and CPUE (kg/km^2^) distribution maps of commercially important demersal fishes (annular seabream *Diplodus annularis*, spotted flounder *Citharus linguatula*) and invertebrates (deep water rose shrimp *Parapenaeus longirostris*). The maps were generated using ArcGIS 10.4.1.5686 (www.esri.com). Fish and invertebrate drawings are by Louizos Verdaris.

The mean total length (L_MEAN_) of commercial fishes and crustaceans was generally higher in the low pressure area compared to the other areas, i.e. L_MEAN_ increased with declining fishing pressure. The L_MEAN_ of red mullet (*Mullus barbatus*), common pandora (*Pagellus erythrinus*), axillary seabream (*Pagellus acarne*), annular seabream (*Diplodus annularis*) and spotted flounder (*Citharus linguatula*) was highest in the low pressure area, while the L_MEAN_ of the deep water rose shrimp (*Parapenaeus longirostris*) was higher in the intermediate compared to the high pressure area, but the difference was not statistically significant (Table 1, Figs 2 and 3).

**Table 1:** Mean length (L_MEAN_) of commercial and non-commercial demersal fishes (total length, mm), and crustaceans (carapace length, mm) per fishing pressure area (low: low pressure; intermediate: intermediate pressure; high: high pressure) and the statistical results of comparing mean lengths (ANOVA) and length frequency distributions (Kolmogorov-Smirnov). Bold in species name denotes statistical difference with area.

Species	Low	Intermediate	High	L_MEAN_ comparison	Length distribution comparison
**Fishes**					
**Commercial demersal**					
***Mullus barbatus***	170	149	155	F = 54.08, P < 0.001	KS = 3.94, P < 0.001
***Pagellus erythrinus***	173	155	-	F = 48.18, P < 0.001	KS = 3.05, P < 0.001
***Pagellus acarne***	094	085	-	F = 17.24, P < 0.001	KS = 2.86, P < 0.001
***Diplodus annularis***	139	117	-	F = 31.06, P < 0.001	KS = 2.53, P < 0.001
***Citharus linguatula***	154	134	108	F = 20.82, P < 0.001	KS = 2.49, P < 0.001
**Non-commercial demersal**					
***Serranus hepatus***	103	091	094	F = 37.52, P < 0.001	KS = 2.63, P < 0.001
*Spicara maena*	119	119	119	F = 0.01, P = 0.99	KS = 0.74, P = 0.64
*Chelidonichthys lastoviza*	173	179	-	F = 1.10, P = 0.29	KS = 1.32, P = 0.06
**Crustaceans**					
*Parapenaeus longirostris*	—	023	019	F = 2.66, P = 0.11	KS = 1.68, P = 0.006

**Figure 4:**
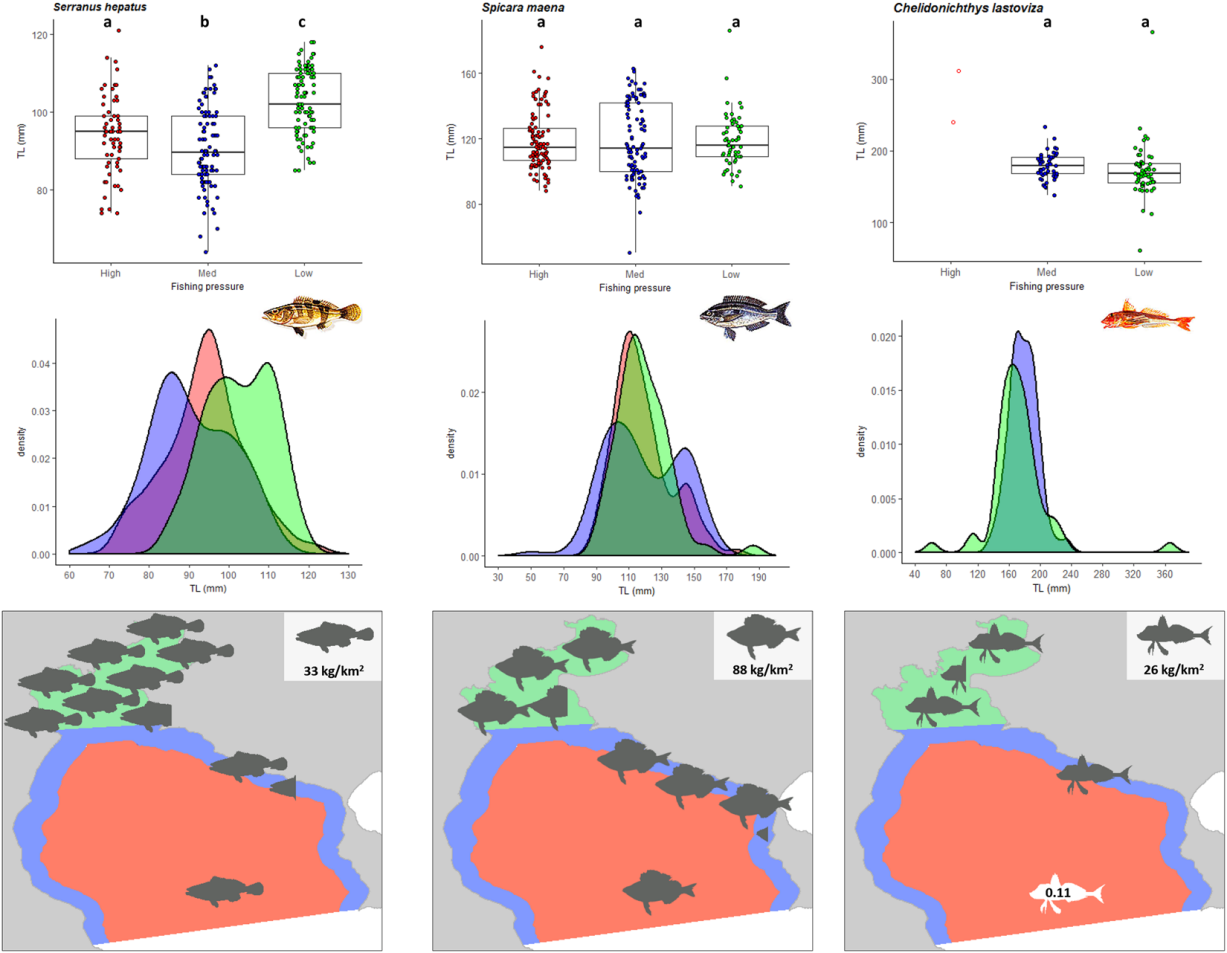
Box plots (total length, mm), density plots of length distribution, and CPUE (kg/km^2^) distribution maps of non-commercial demersal fishes (brown comber *Serranus hepatus*, blotched picarel *Spicara maena*, streaked gurnard *Chelidonichthys lastoviza*). The maps were generated using ArcGIS 10.4.1.5686 (www.esri.com). Fish drawings are by Louizos Verdaris.

The L_MEAN_ of non-commercial fish species either did not differ among pressure areas or was higher in the low pressure area. The L_MEAN_ of brown comber (*Serranus hepatus*), a species that is caught and discarded, was higher in the low fishing pressure area compared to the other two areas (Table 1, Fig. 4), while the L_MEAN_ of the other two non-commercial fishes (blotched picarel *Spicara maena* and streaked gurnard *Chelidonichthys lastoviza*) did not differ among areas (Table 1, Fig. 4).

Similarly to the comparison of L_MEAN_, the length frequency distributions of commercial fishes were also different between high and low pressure areas (Table 1) with the density plots of all species shifted towards higher sizes in the low pressure area and towards lower sizes in the high pressure area (Figs 2 and 3). With the exception of brown comber that followed the pattern of commercial fishes (Table 1), the length distributions of the non-commercial species (Table 1) did not differ between high and low fishing pressure areas (Fig. 4).

### Population biomass

The mean CPUE of commercial fishes and invertebrates was lowest in the high pressure area. The highest CPUE of red mullet was recorded in the low pressure area (149 kg/km^2^) compared to the intermediate and high pressure areas (105 kg/km^2^, for both) (Fig. 2). The CPUE of common pandora was higher in the intermediate pressure area (106 kg/km^2^) compared to the low (24 kg/km^2^) and high pressure areas (1 kg/km^2^) (Fig. 2). The same pattern was observed for the spotted flounder with higher CPUE in the intermediate pressure area (75 kg/km^2^) compared to the low (40 kg/km^2^) and high pressure areas (14 kg/km^2^) (Fig. 3). The CPUE of the axillary seabream was much higher in the low pressure area (524 kg/km^2^) compared to the intermediate (165 kg/km^2^) and high pressure areas (0.4 kg/km^2^) while the CPUE of annular seabream was higher in the low pressure area (256 kg/km^2^) compared to the intermediate (22 kg/km^2^) (Figs 2 and 3). Finally, the CPUE of deep water rose shrimp was higher in the high pressure area (9 kg/km^2^) compared to the intermediate (1.3 kg/km^2^) (Fig. 3).

The mean CPUE of all non-commercial fishes was highest in the low fishing pressure area and lowest in the high fishing effort area. The CPUE of brown comber was very high (250 kg/km^2^) in the low fishing pressure area compared to the intermediate and high pressure areas, which were low in CPUE, 45 kg/km^2^ (intermediate pressure) and 33 kg/km^2^ (high pressure) (Fig. 4). The CPUE of the blotched picarel was much higher in the low and intermediate pressure areas (320 kg/km^2^ and 288 kg/km^2^, respectively) compared to the high pressure area (88 kg/km^2^), while the CPUE of streaked gurnard was double in the low (61 kg/km^2^) compared to the intermediate (26 kg/km^2^) pressure area (Fig. 4).

### Length indicators

The maximum length (L_MAX_) of commercial fishes and crustaceans was generally higher in the low pressure area and declined with increasing fishing pressure. The L_MAX_ of red mullet exhibited a different pattern with the highest value in the intermediate pressure area (L_MAX_ = 222 mm) and the lowest in the low pressure area (L_MAX_ = 200 mm), while that of common pandora, axillary seabream and annular seabream was highest in the low pressure area (Figs 2 and 3). The L_MAX_ of the spotted flounder was lowest in the high pressure area (L_MAX_ = 222 mm), and, finally, the L_MAX_ of the deep water rose shrimp was higher in the high (L_MAX_ = 34 mm) compared to the intermediate pressure area (L_MAX_ = 31 mm, Fig. 3).

The L_2/3_ of commercial species was also higher in the low pressure area and declined with increasing pressure, with the exception of deep water rose shrimp that had similar L_2/3_ in intermediate and high pressure areas (Table 2). The same trend was also observed in non-commercial fishes, except for the blotched picarel that had a higher L_2/3_ in the intermediate pressure area (Table 2).

**Table 2:** The percentage of individuals greater than 2/3 of the maximum total length (L_2/3_), the 95% percentile of the length distribution (L_0.95_), and the L_MAX_/L_SUPREME_ ratio for the demersal species per fishing pressure area (low: low pressure; intermediate: intermediate pressure; high: high pressure). Fishes: total length (mm); crustaceans: carapace length (mm). L_MAX_ is the maximum length recorded per species during the survey and L_SUPREME_ is the maximum length ever recorded for each species in the Mediterranean according to FishBase^61^.

	L_2/3_			L_0.95_			L_MAX_/L_SUPREME_		
Species	Low	Intermediate	High	Low	Intermediate	High	Low	Intermediate	High
**Fishes**									
**Commercial demersal**									
*Mullus barbatus*	0.95	0.38	0.5	187	197	188	0.53	0.58	0.56
*Pagellus erythrinus*	0.07	0.01	—	203	187	—	0.50	0.35	—
*Pagellus acarne*	0.44	0.10	—	118	99	—	0.39	0.38	—
*Diplodus annularis*	0.89	0.29	—	168	131	—	0.75	0.58	—
*Citharus linguatula*	0.17	0.10	0.01	208	212	164	0.74	0.92	0.64
**Non-commercial demersal**									
*Serranus hepatus*	1.00	0.84	0.88	115	106	110	0.47	0.45	0.48
*Spicara maena*	0.31	0.42	0.29	142	154	149	0.62	0.54	0.59
*Chelidonichthys lastoviza*	0.02	0.00	—	218	204	—	0.92	0.59	—
**Crustaceans**									
*Parapenaeus longirostris*	—	0.13	0.14	—	28	29	—	0.60	0.66

With the exceptions of red mullet and deep water rose shrimp, the L_0.95_ of commercial species was also higher in the low pressure area and declined with increasing pressure (Table 2); the same trend was also observed in non-commercial fishes, except for the blotched picarel (Table 2).

The L_MAX_/L_SUPREME_ ratio was generally increasing with declining pressure for commercial fishes with the exception of red mullet and deep water rose shrimp (Table 2). It was also not related to fishing pressure for the non-commercial fish species. Overall, the lowest L_MAX_/L_SUPREME_ ratio, i.e. the largest divergence from virgin population size structure was observed for axillary seabream and common pandora.

### Length at maturity

**Figure 5:**
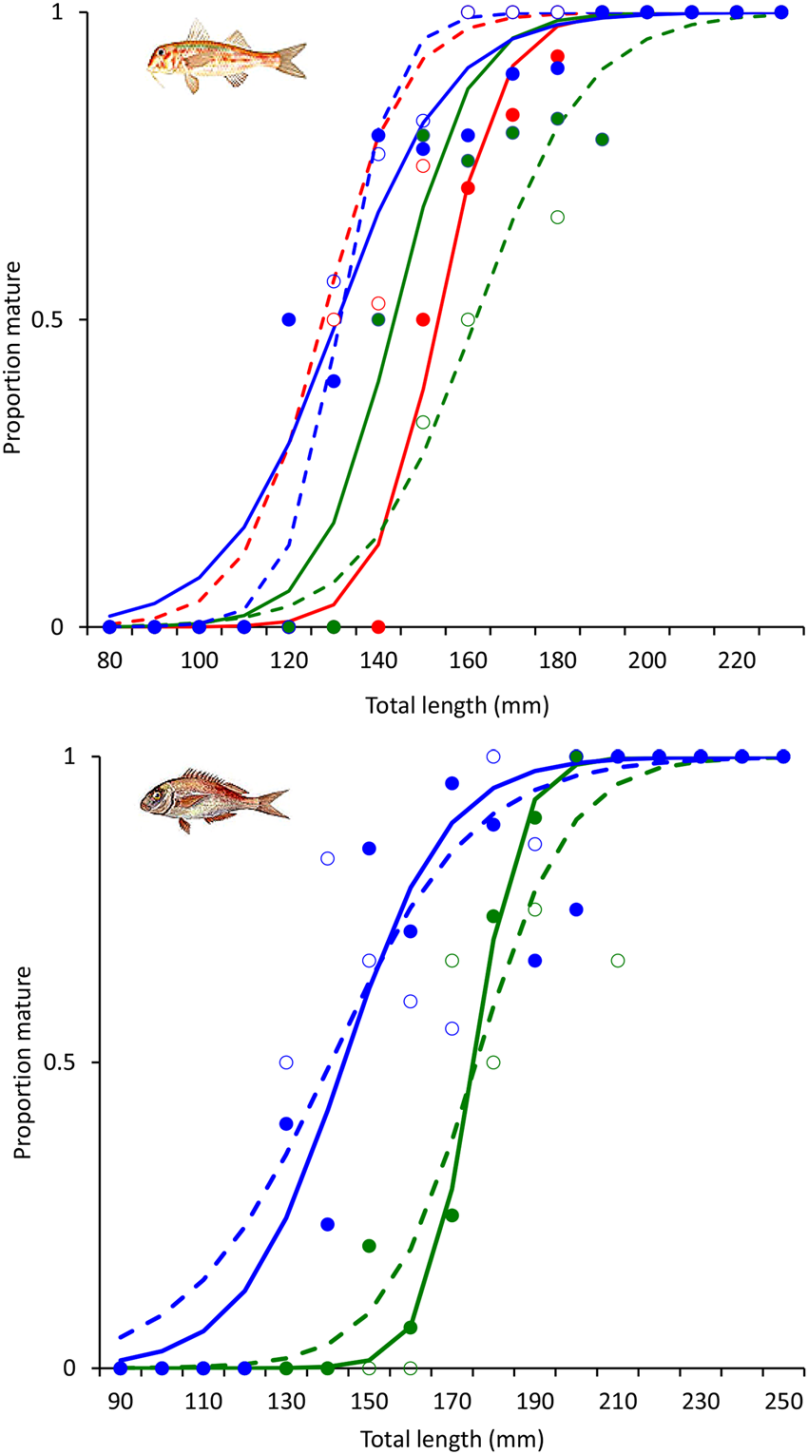
Observed (circles) and modeled (lines) lengths (total length, mm) at maturity for males (open circles, dashed lines) and females (solid circles, continuous lines) of red mullet (*Mullus barbatus*, top panel) and common pandora (*Pagellus erythrinus*, bottom panel) among the pressure areas (green: low fishing pressure; blue: intermediate fishing pressure; red: high fishing pressure). Fish drawings are by Louizos Verdaris.

Length at maturity (L_M_) was estimated for both males and females of red mullet and common pandora and was compared among pressure areas (Fig. 5, Table 3).

**Table 3:** Length at maturity (L_M_; total length, mm) for females and males of red mullet (*Mullus barbatus*) and common pandora (*Pagellus erythrinus*) per fishing pressure area (low: low pressure; intermediate: intermediate pressure; high: high pressure).

Area		Red mullet	Common pandora
**Low**	Females	144	175
	Males	162	176
**Intermediate**	Females	131	144
	Males	131	141
**High**	Females	153	—
	Males	128	—

For red mullet (present in all areas), size at maturity exhibited a different pattern between sexes (Fig. 5, top panel; Table 3). Size at maturity was lowest for males in the high pressure area and highest in the low pressure area. For females, L_M_ was lowest in the intermediate pressure area and highest in the high pressure area.

For common pandora (present only in the intermediate and low pressure areas) the maturity pattern was the same for males and females with respect to fishing pressure (Fig. 5, bottom panel; Table 3). Size at maturity was 30–35 mm lower for both sexes in the intermediate pressure area compared to the low pressure one.

## Discussion

The results of this study, based on somatic length (L_MEAN_, L_MAX_, L_2/3_, L_0.95_, L_MAX_/L_SUPREME_) and CPUE indicators showed that, in general, the size structure and biomass of demersal fish and invertebrate populations are in better condition in areas where fishing pressure is lower due to permanent fishing restrictions. Despite some contradicting studies, numerous researchers have shown the positive effects of permanent spatial protection on marine populations. Indicatively, Dugan and Davis^16^ reviewed about thirty studies on protected areas incorporating fishing restrictions, and found that most of them reported increased abundances (density or biomass), as well as greater mean individual sizes (lengths or weights) of target species inside refuges compared to adjacent fished areas.

Fishing is the human activity that most heavily impacts the seabed and directly affects demersal fish populations and communities^2^. In addition to direct impacts on the mortality of marine populations, fishing can modify their population structure and size composition^24^, and thereby affect marine food webs and ecosystems^26^. Given that fishing is a size selective process and that maximum somatic length is a good proxy for life-history characteristics^27^ and species sensitivity to mortality^28,29^, the concept of using size as a proxy for sensitivity to fishing impacts is well grounded in theory^30^. Indeed, there are several size-based indicators that are able to respond to the effects of fishing^31^ and indicate the health of a stock, even in the presence of confounding factors not related to fishing^32^. The health of the stock increases as the age and size distribution consists of more, older and larger fish and the length distribution of the population resembles that in its virgin state, or at least when fishing exploitation was low^33^. Therefore, permanent restrictions on fishing activity are generally improving the health of the stocks by protecting their spawning biomass and nursery habitats^34^, allowing recruitment to the adult stock^35^, and fish somatic growth, thus preserving length and age distribution^36^. They also provide a refuge for large individuals and their important contribution to the gene pool, which is lost in the fished area proportional to fishing effort^37^.

All commercial species in Thermaikos Gulf were larger in size within the low fishing pressure area and their size was declining with increasing fishing pressure, whereas the non-commercial species generally did not differ with varying fishing pressure, except for the brown comber that is commonly caught and discarded by trawlers (Table 1). Similar results have been reported in the SE North Sea, with the marketable sizes of the commercial fish species increasing in abundance as the fishing effort was reduced, whereas the size structure of non-commercial fish species was not affected^38^. Likewise, in a Mediterranean reef assemblage, the largest individuals of all species vulnerable to fishing were more abundant in the reserve area^39^. Also, in SW Portugal, the number of larger fish specimens of commercially important species increased over time in areas where almost all fishing activities were forbidden^8^. In southern California, the largest size classes of temperate legal-sized targeted fish species were observed only inside marine reserves, in increased densities and biomasses^37^. In the present work, only red mullet diverged from the above-mentioned pattern in the intermediate and high pressure areas due to the intense exploitation of the species by the coastal fleet (mainly netters that operate throughout the year) along the coast (intermediate pressure area) and by the trawlers operating in deeper waters, further offshore (high pressure area).

The contrasting effect of fishing between commercial and non-commercial species has been reported in the scientific literature since the late 19^th^ century. In a rather heretic view, Fulton^1^ maintains that “*commercial trawling can positively affect commercially unimportant fish*” because the catch rates for abundant not commercially important species, which are caught and discarded, were higher in the areas open to fishing. Similarly, the abundance of the demersal fish assemblage and Argentine hake (*Merluccius hubbsi*) was higher in the area that is closed to any type of fishing, whereas for non-target species the abundance was higher in the adjacent fished areas^40^ where predators, not benefiting from any protection measure, are removed by fishing^41^. In the present study, some of the non-commercial species that are common by-catch of trawlers (brown comber) were affected the same way the commercial species are, but others were less impacted by fishing because they are less vulnerable to the trawling gear (as is the case for the blotched picarel) and invulnerable to nets that target specific stocks. In addition, the non-commercial species are not targeted by local fishers who may modify their tactics and change fishing grounds to avoid areas with a lot of unwanted and unmarketable catch.

In other cases, species that are targeted by commercial fisheries have been shown to be more abundant within the boundaries of protected areas, whereas non-target ones seem to be as abundant as outside of marine reserves^42^. In southern California, targeted temperate fish species showed higher mean density, size and biomass inside marine reserves, whereas the density of non-targeted species exhibited no difference between protected and not protected areas, thus corroborating the observed patterns being attributed to fishing pressure rather than site-specific impacts^37^, as was the case in the present study. In the SE North Sea, fish density was observed to be higher in the area with reduced fishing effort, for both commercial and non-target species^38^.

Here, the comparison of length based indicators and length frequency distributions among varying fishing pressure areas confirmed that, at the population level, the decline of length and the shifted distributions to lower length classes reflect the impact of fishing^42^. Indeed, the high fishing pressure imposed on small individuals and the reduced probability of them surviving to larger sizes causes the strong decline in large individuals^43^ that disrupts population structure and causes declines in mean size both within and among species^44^. The decline of larger individuals/species has further implications for future fisheries catches because smaller individuals may suffer higher natural mortalities as they are more susceptible to predation^28^.

The presence of immature individuals of red mullet in the inner and coastal areas of Thermaikos Gulf (Fig. 2) indicates that these areas serve as nursery ground for the species. Similarly, Piet and Rijnsdorp^38^ found the small sized fish of the commercially exploited species in the area closed-to-fishing. In European Mediterranean waters, the only nurseries consistently protected are those of coastal species, such as red mullet (67% of nursery area protected) and common pandora (54%), with this protection regime stemming from the trawling ban within 3 nautical miles off the shoreline or 50 m depth (Article 13 of EU Council Regulation 1967/2006), whereas for species with offshore recruitment areas such as European hake (*Merluccius merluccius*) and the deep-water rose shrimp, the proportion of nursery areas protected is lower and inconsistent^45^. Although the vertical distribution of the deep water rose shrimp is wide (20–700 m depth), the species is mostly encountered between 100 and 400 m depth with the juveniles settling in shallower waters and the larger individuals having a deeper than 350 m distribution^46^. Hence, the biology of the species corroborates our findings regarding the higher biomass in the deeper hauls (high pressure area), but also the non-significant difference in the mean length of the individuals, as the trawling ban in the shallow Thermaikos Gulf cannot offer protection to the - commercially desirable - largest individuals of the species that inhabit much deeper waters.

Red mullet and common pandora, two of the main target species of most fishing fleets and gears, exhibited different maturation patterns among the fishing pressure areas and generally matured at smaller length in high pressure areas and at larger length in low fishing pressure areas. The intensity of fishing pressure has been shown to affect the maturation of fish stocks causing earlier maturation for stocks that suffer higher fishing mortality rates^47^. Earlier maturation is considered as an evolutionary response to high fishing pressure^48^ aiming to increase the probability of a stock surviving to spawn and to reduce generation time^49^. However, earlier maturation can lead to reduced fecundity because small-sized individuals produce less oocytes and are prone to natural mortality due to the higher levels of predation they experience^28^. In order for the stock biomass to be maintained, fish should be allowed to spawn at least once over their lifespan before being caught^50^, which premises that immature fish should not be caught^51^.

## Conclusions

Fishers are generally skeptical about spatial restrictions worrying that limiting their fishing grounds, through spatial fishing restrictions, will lead to decreased catches and longer travelling time to the fishing ground^4^ and therefore reduced earnings. Although they generally seem to accept the restrictions that serve fisheries management purposes and especially the more flexible (e.g. gear-restriction zones, temporal area closures) over the more restrictive (e.g. no-take MPAs) ones, they need to be provided with evidence of the benefits of such restrictions at a local scale^52^. The results of this study can serve as sufficient and convincing justification for fishers, managers and other local stakeholders that permanent fishing restrictions benefit the size structure and biomass of commercial demersal fish within their boundaries. And since the illusion that more fishing equals higher catches and higher profit is well outdated^53^, the benefits of fishing restrictions on commercial fish stocks in fact benefit the fishers themselves^7^ and act as insurance for the stock against loss of important genes.

## Materials and Methods

### Study area

Thermaikos Gulf is a shallow gulf in the northwestern Aegean Sea covering an area of around 3340 km^2^ (according to the official definition of Thermaikos Gulf, including Thessaloniki Gulf and Bay: Presidential Decree 189/1978; see also supplementary material) with a maximum depth not exceeding 100 m (Fig. 1).

Thermaikos Gulf is one of the most important fishing grounds in the northeastern Mediterranean Sea, with the second highest fishing effort of trawlers and purse seiners in the Aegean Sea^54^. The catches from Thermaikos Gulf account for 25% of the total Greek catches and mainly consist of European anchovy (*Engraulis encrasicolus*) and European pilchard (*Sardina pilchardus*) for purse seiners, European hake, red mullet, surmullet (*Mullus surmuletus*), and deep water rose shrimp for trawlers, as well as common cuttlefish (*Sepia officinalis*), common octopus (*Octopus vulgaris*) and crabs for coastal vessels and trawlers combined^55^.

Based on spatial and temporal fishing restrictions, the study area is divided in three areas of varying fishing pressure (Fig. 1). Its innermost part (Thessaloniki Bay and Gulf, and inner Thermaikos Gulf; low fishing pressure area; green in Fig. 1) is protected from large-scale fishing activities, as bottom trawl and purse-seine fishing have been totally banned since 1978^56^ and small-scale coastal vessels operate seasonally (see supplementary material). The coastal part of the outer Thermaikos Gulf (intermediate fishing pressure area; blue in Fig. 1) is protected from large-scale fishing activities, as operating with bottom trawls and purse-seines is prohibited but small-scale coastal vessels operate throughout the year using several fishing gears^56^ (see supplementary material). Finally, the open water part of the outer Thermaikos Gulf (high fishing pressure area; red in Fig. 1) is being exploited by all fishing gears (see supplementary material).

### Sampling design

The sampling scheme was designed to include two stations in each of the fishing pressure areas that are subjected to varying fishing effort. Six bottom trawl hauls were conducted in Thermaikos Gulf during summer 2016 (Fig. 1) onboard a commercial trawler that was hired for experimental purposes. The gear used was the same experimental bottom trawl that is being used during the MEDITS survey^57^. The hauling duration was the same (30 min) in all stations because of the shallow depth of the entire sampling area. The substrate was characterized by mud, sandy mud and muddy sand sediments across the study area^54,58^.

Two of the hauls (L1 and L2) were located inside the fisheries restricted area (low fishing pressure area; Low) and two hauls (M3 and M4) were located in areas with intermediate fishing pressure (Med), where coastal vessels, using nets and longlines, operate throughout the year (Fig. 1; see also supplementary material). It should be noted here that these hauls are on the boundaries of areas allowed for trawling where extensive illegal activity (mainly trawling), violating the spatial restrictions, is being recorded, especially near haul M3, based on vessel monitoring systems (www.globalfishingwatch.org). Finally, two hauls (H5 and H6) were located in the high fishing pressure areas (High), where coastal vessels, using nets and longlines, operate throughout the year and the majority of the trawling fleet operate during their fishing season (Fig. 1; see also supplementary material).

### Data collection

The biomass (kg) of all fish and invertebrate species was recorded onboard. Total length (mm) was measured for all individuals of target fish species and carapace length (mm) was measured (referred as total length) for crustaceans. Sex and maturity stages were also determined for the target species, which were four fishes, European hake, red mullet, surmullet, common pandora and two crustaceans, Norway lobster (*Nephrops norvegicus*) and deep water rose shrimp, as well as sharks and rays. European hake, surmullet, Norway lobster, sharks and rays were excluded from the analyses because of the low number of individuals caught. However, the analyses included three abundant commercially important demersal species, initially not included among the target species (axillary seabream, annular seabream, spotted flounder) and three non-commercial species that often occur as by-catch (brown comber, blotched picarel, streaked gurnard). Brown comber is discarded at high rates by several gears.

### Data analysis

#### Size structure

The adult length structure of the populations across areas of varying fishing pressure was compared using the mean lengths and length frequency distributions that were constructed for the nine species for which data were available. Density plots (an unbounded, continuous and smoothed version of the histogram) were preferred to length frequency distribution histograms for clarity in presentation. To assess differences in size of adults of the target species, mean total lengths (mm) of individuals were compared across areas using ANOVA, testing the hypothesis of no length difference among areas. One-way ANOVA and multiple range tests were performed to determine whether the mean lengths of the three areas are different from each other^59^. The length frequency distributions between high and low fishing pressure areas were compared using the Kolmogorov-Smirnov (KS) test. When recorded, as in the case of red mullet, early juveniles were treated separately and were excluded from the mean length and frequency distribution comparison of adults among areas. Yet, the red mullet juveniles appear in the box plots because their presence provides important information denoting nursery and potential spawning habitat for the species in Thermaikos Gulf.

#### Population biomass

The population biomass per species was expressed as catch per unit of effort (CPUE, in kg/km^2^) given that the duration of the hauls was the same across stations. The duration was 30 min as all hauls were considered shallow, i.e. shallower than 200 m, and the swept area was determined based on the distance covered by the haul and the gear characteristics^57^.

#### Length indicators

Three indicators that are related to population size and age distribution and provide information on the effects of fisheries exploitation on a stock^30^ were also computed per area for the demersal species:

- The percentage of individuals greater than 2/3 of the L_MAX_ (L_2/3_), which shows the proportion of large individuals and how length structure is affected by fishing,
- The 95% percentile of the fish length distribution (L_0.95_), which is expected to be sensitive to fishing and other human impacts^60^,
- The dimensionless ratio of L_MAX_ to the highest value of length ever recorded for the species in the Mediterranean (L_SUPREME_), L_MAX_/L_SUPREME_, which measures the divergence from virgin population size structure and shows the historical fishing pressure the population in each area has been subjected to. Stock specific L_SUPREME_ values for the Mediterranean Sea were extracted from FishBase^61^.

These indicators require data from research surveys but they can also be computed based on commercial catch data. In the present work, they were computed for comparing the areas of varying fishing pressure, but they can also be used as reference points given that the low fishing pressure area has not been fished with mobile bottom gear for 40 years.

#### Length at maturity

Length at maturity (i.e. length at which 50% of individuals attained sexual maturity, L_M_) was determined for the species with adequate sample size that would allowed a comparison among areas for both males and females (red mullet and common pandora) by fitting a logistic curve to the percentage of mature fish (P) per 0.5 cm total length (L)^62^.

1 $$P=\frac{{e}^{({v}_{1}+{v}_{2}L)}}{(1\,+{e}^{({v}_{1}+{v}_{2}L)})}$$

and

2 $${L}_{M}=-\frac{{v}_{1}}{{v}_{2}}$$

where v_1_ and v_2_ determine the intercept and slope of the logistic curve.

### Experiments including animals

The trawling survey conducted in the present study was licensed by the Greek Government (Director General of the Decentralised Administration of Macedonia-Thrace) to the Hellenic Centre for Marine Research (HCMR) for the purposes of the European research program “Towards the establishment of Marine Protected Area Networks in the Eastern Mediterranean – PROTOMEDEA” in Thermaikos Gulf (Document number 45.037, 5/7/16, available upon request in Greek). All sampling methods were carried out in accordance with relevant guidelines and regulations (Law 1197/1981).

### Data availability

The datasets generated and analysed during the present work are available from the corresponding author on reasonable request.

## Electronic supplementary material

Supplementary material
