# Supplementary material for: Spatial fishing restrictions benefit demersal stocks in the northeastern Mediterranean Sea
Source: Sci Rep. 2018 Apr 13;8:5967. doi: 10.1038/s41598-018-24468-y (PMC5899153; doi:10.1038/s41598-018-24468-y)
Supplement: Supplementary file 1 — Supplementary material [file 41598_2018_24468_MOESM1_ESM.docx]

**Supplementary material to**

**“Spatial fishing restrictions benefit demersal stocks in the northeastern Mediterranean Sea”**

Donna Dimarchopoulou^1^, Aikaterini Dogrammatzi^2^, Paraskevi K. Karachle^2^, Athanassios C. Tsikliras^1^

^1^Laboratory of Ichthyology, Department of Zoology, School of Biology, Aristotle University of Thessaloniki, 54124 Thessaloniki, Greece

^2^Institute of Marine Biological Resources and Inland Waters, Hellenic Centre for Marine Research, 16604 Attica, Greece

Email: ddimarch@bio.auth.gr

**Fisheries regulations in Thermaikos Gulf**

According to the fleet registry^1^, over 1450 fishing vessels were registered in the study area in 2014, 58 of which were trawlers (using bottom trawls, OTB), 29 were purse seiners (using purse-seines, PS) and around 1370 were small-scale coastal vessels using a wide variety of fishing gears. European hake (*Merluccius merluccius*), red mullet (*Mullus barbatus*), and deep water rose shrimp (*Parapenaeus longirostris*) constitute the main target species of trawl fishing in the eastern Mediterranean^2^.

The activities of fishing vessels (trawlers, purse seiners, and small-scale coastal vessels) and recreational fishing in Thessaloniki and Thermaikos Gulfs (Supplementary Figure S1) are regulated by the Presidential Decree (P.D.) 68/2009 (Efimeris tis Kyverniseos No. 90, Part I, 12 June 2009, pp. 5127-5130) which is entitled “Regulation of fishing in the gulfs of Thessalonica and Thermaikos”. Unless otherwise stated in the P.D. 68/2009, the general fisheries regulations according to the European legislation, are valid across the Greek seas. P.D. 68/2009 replaced the older P.D. 189/1978 (Efimeris tis Kyverniseos No. 41, Part I, 24 March 1978, pp. 331-333) with the same title.

According to the P.D. 68/2009, bottom trawling is totally prohibited in Thessaloniki Bay, Thessaloniki Gulf and inner Thermaikos Gulf, while in outer Thermaikos Gulf bottom trawling is prohibited within 3 nautical miles from coastline or within the 50 m depth contour should that depth be reached at shorter distance (i.e. <3 nm) from the coastline; within 2 nautical miles from the coastline regardless sea depth (Supplementary Figures S1 and S2); bottom trawling is also prohibited over seagrass beds (art. 3 of the P.D. 68/2009). Trawling is prohibited within 3 nautical miles from river mouths throughout the year (art. 10) and within a radius of 50 m around permanent bivalve aquaculture units (art. 10). Bottom trawls operate from 1^st^ of October to 24^th^ of May, inclusive. Trawlers and purse-seiners operate throughout the year in international waters, defined as 6 nautical miles from shore, except for trawlers from July 16th to October 1^st^ (Supplementary Figure S2).

Purse-seining is totally prohibited in Thessaloniki Bay and Gulf, while in Thermaikos Gulf purse-seining is prohibited within 300 m from coastline or within the 50 m depth contour should that depth be reached at shorter (<300 m) distance from the coastline (art. 4) (Supplementary Figure S3). Purse-seining is prohibited within a radius of 50 m around permanent bivalve aquaculture units (art. 10). Purse-seines operate from March to the 15th of December but not two days before, during and two days after the full moon (Supplementary Figure S3).

Small-scale coastal vessels operate throughout the year using various gears. The minimum stretched bar length for nets is set at 36 mm, except for sardine nets in which it is set at 16 mm (art. 5). The use of nets with stretched bar length below 44 mm is prohibited in Thessaloniki Gulf from the 10th of July to the 10th of September each year (art. 5). The use of nets with stakes («*kalamota*») is totally prohibited both in Thessaloniki and Thermaikos Gulfs in June and July each year (art. 5). Caramote prawn (*Melicertus kerathurus*) fishing with nets is also prohibited between the 10th of July to the 10th of September each year (art. 5). Netting is prohibited throughout the year at depths shallower than 2 m (art. 10) and within a radius of 50 m around permanent bivalve aquaculture units (art. 10).

Fishing with light is totally prohibited in Thessaloniki Gulf, while caramote prawn fishing with light in Thermaikos Gulf is prohibited between January 1^st^ and July 10^th^, and between the 11^th^ and the 30^th^ of September (art. 6). Only one source of light per vessel is allowed when fishing caramote prawn with light in Thermaikos Gulf (art. 6).

Fishing with traps is totally prohibited in Thessaloniki Gulf and part of Thermaikos Gulf (art. 7). Each vessel using traps is allowed to carry 500 pairs of fykenets or 300 traps. The use of fykenets is prohibited in Thessaloniki and Thermaikos Gulfs in July and August each year (art. 7). Setting the fykenets at depths below 2 m is prohibited throughout the year (art. 7) and within a radius of 50 m around permanent bivalve aquaculture units (art. 10).

Semi-permanent nets in the sea can be set at depths from 6 to 12 m and at distance exceeding 2000 m among them (art. 8). The stretched bar length of the gill-nets used cannot be below 20 mm. No other fishing gear is allowed to operate within 200 m from semi-permanent net installations.

Regarding recreational fishing, the maximum allowed quantity should not exceed 3 kg of fish and cephalopods per 24h per fishing boat or per fisher when fishing from shore (art. 9). Spearfishing, with or without light, is completely prohibited in Thessaloniki and Thermaikos Gulfs throughout the year (art. 9).

All fishing activities are prohibited in Thessaloniki Bay (Supplementary Figure S1).

**References**

1. CFR (2014) European Commission 2014. Common Fisheries Registry (Accessed 07/03/2014).
2. Stergiou, K.I., Moutopoulos, D.K. & Tsikliras, A. C. Spatial and temporal variability in Hellenic marine fisheries landings in *State of Hellenic Fisheries* (eds. Papaconstantinou, C., Zenetos, A., Vassilopoulou, V. & Tserpes, G.) 141-150 (Hellenic Centre for Marine Research, 2007).


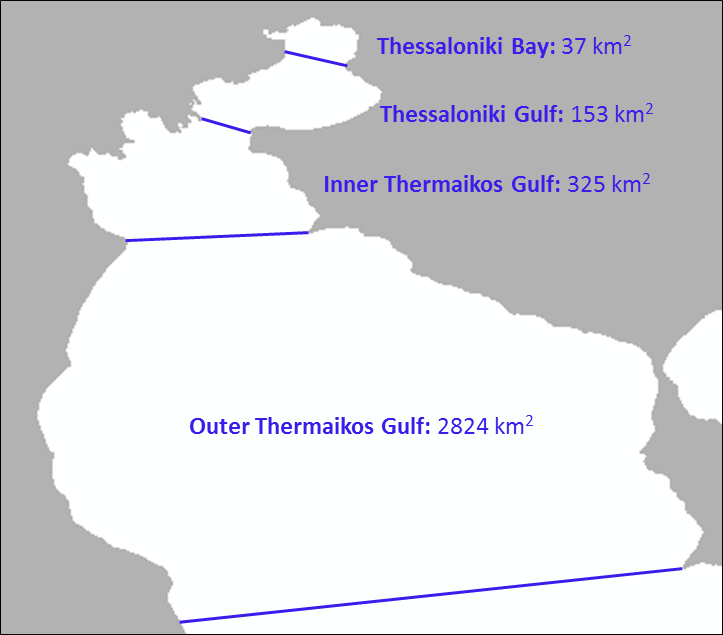


**Supplementary Figure S1.** Map of the broader Thermaikos area. The map was generated using ArcGIS 10.4.1.5686 (www.esri.com).


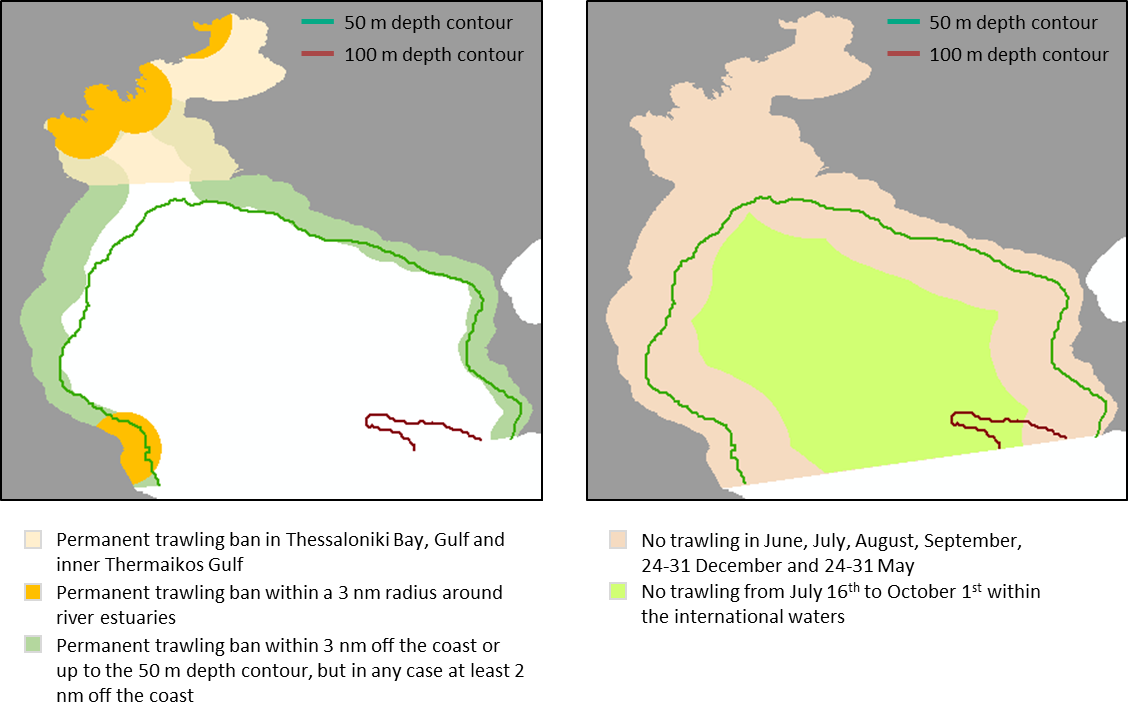


**Supplementary Figure S2.** Map of the study area (Thermaikos Gulf; NW Aegean Sea; NE Mediterranean Sea) depicting spatial permanent (left) and seasonal (right) trawling bans as reported in Presidential Decree 68/2009. The maps were generated using ArcGIS 10.4.1.5686 (www.esri.com).


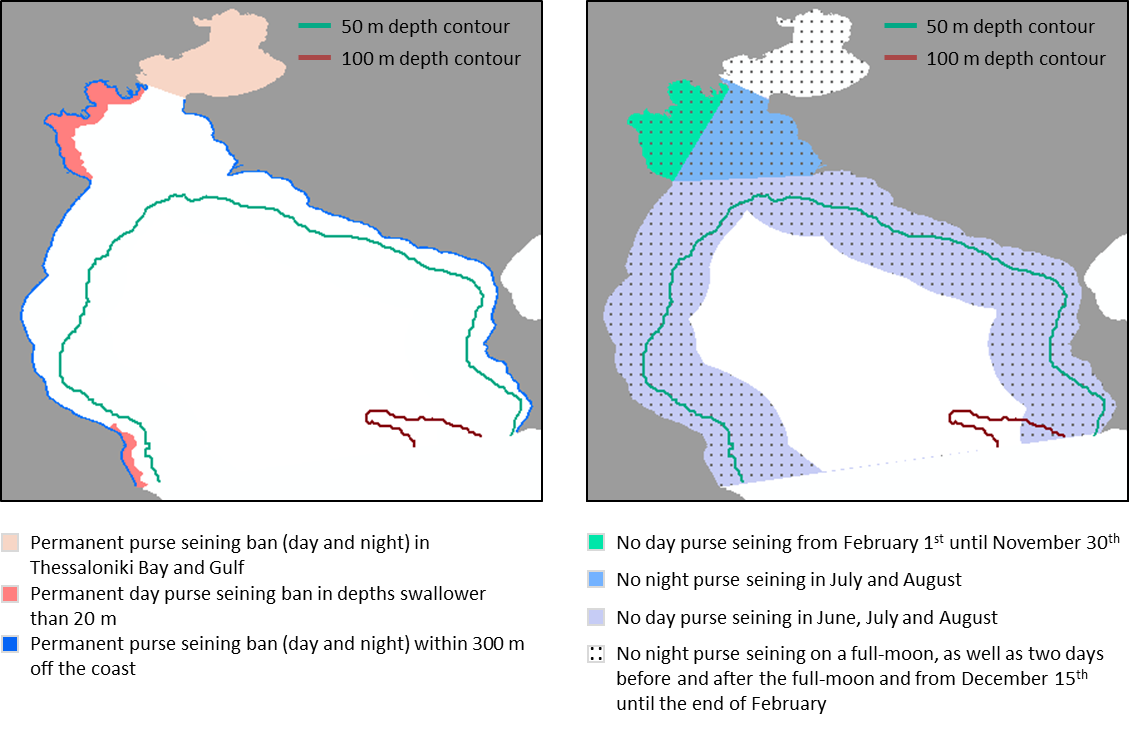


**Supplementary Figure S3.** Map of the study area (Thermaikos Gulf; NW Aegean Sea; NE Mediterranean Sea) depicting spatial permanent (left) and seasonal (right) purse seining bans as reported in Presidential Decree 68/2009. The maps were generated using ArcGIS 10.4.1.5686 (www.esri.com).
